# Integrating 400 million variants from 80,000 human samples with extensive annotations: towards a knowledge base to analyze disease cohorts

**DOI:** 10.1186/s12859-015-0865-9

**Published:** 2016-01-08

**Authors:** Jörg Hakenberg, Wei-Yi Cheng, Philippe Thomas, Ying-Chih Wang, Andrew V. Uzilov, Rong Chen

**Affiliations:** Department of Genetics and Genomic Sciences, Icahn School of Medicine at Mount Sinai, 1425 Madison Ave, Box 1498, New York, 10029 USA; Department of Computer Science, Humboldt-Universität zu Berlin, Unter den Linden 6, Berlin, 10099 Germany; Current affiliation: Illumina, Inc., 451 El Camino Real, Suite 210, Santa Clara, 95050 USA; Current affiliation: Roche Parma Research and Early Development, Informatics, Roche Innovation Center New York, 430 East 29th St, New York, 10016 USA; Current affiliation: German Research Centre for Artificial Intelligence (DFKI), Alt Moabit 91c, Berlin, 10559 Germany

**Keywords:** Genetics, Variant annotation, Database

## Abstract

**Background:**

Data from a plethora of high-throughput sequencing studies is readily available to researchers, providing genetic variants detected in a variety of healthy and disease populations. While each individual cohort helps gain insights into polymorphic and disease-associated variants, a joint perspective can be more powerful in identifying polymorphisms, rare variants, disease-associations, genetic burden, somatic variants, and disease mechanisms.

**Description:**

We have set up a Reference Variant Store (RVS) containing variants observed in a number of large-scale sequencing efforts, such as 1000 Genomes, ExAC, Scripps Wellderly, UK10K; various genotyping studies; and disease association databases. RVS holds extensive annotations pertaining to affected genes, functional impacts, disease associations, and population frequencies. RVS currently stores 400 million distinct variants observed in more than 80,000 human samples.

**Conclusions:**

RVS facilitates cross-study analysis to discover novel genetic risk factors, gene–disease associations, potential disease mechanisms, and actionable variants. Due to its large reference populations, RVS can also be employed for variant filtration and gene prioritization.

**Availability:**

A web interface to public datasets and annotations in RVS is available at https://rvs.u.hpc.mssm.edu/.

**Electronic supplementary material:**

The online version of this article (doi:10.1186/s12859-015-0865-9) contains supplementary material, which is available to authorized users.

## Background

As high-throughput sequencing technologies become more widely employed, variants detected in large resequencing studies are continuously being published, including the 1000 Genomes Project, ESP6500, ExAC, and TCGA [1–4]. These variants differ from the ones targeted by genotyping arrays, in that most of them will initially not be properly annotated with genes, amino acid changes, impacts, associated diseases, or population frequencies. Individual and multi-sample data sets each require exhaustive annotation, using tools such as snpEff, ANNOVAR, or VEP [5–7], predictions of deleteriousness provided by SIFT, PolyPhen2, PROVEAN, and others [8–10] and curated databases such as dbSNP, ClinVar, and HGMD [11–13] to provide as detailed a picture as possible supporting interpretation on a sample-by-sample basis. Notably, for every set of newly called variants, current setups require the annotation of each variant from scratch: even though many variants were observed in earlier studies, aforementioned algorithms and database lookups will be run again on every new call set. Especially the computation of functional predictions and population frequencies are costly and need not be run on recurring variants.

By integrating the results of multiple sequencing efforts, covering a large number of healthy subjects, with such information, we can construct a repository that serves two major purposes: annotating large numbers of genetic variants by aforementioned tools and databases; as well as pooling variants and their frequency distributions in various populations. While the first is primarily aimed at decreasing the operations needed to fully annotate new studies, the second provides a fundamental basis for analyses of disease populations, surpassing the capabilities of each individual study to function as a reference population.

In this paper, our major goal is to build an infrastructure that allows centralized storage of every variant observed in resequencing studies, in-house projects, or known in curated databases. In this centralized storage, variants will be annotated once using a spectrum of tools for functional impact and predictions, as well as population frequencies, diseases-associations, pharmacogenetic information, literature mining, and so on. With each additional sequencing study, the amount of truly novel variants will become less—as shown, for example, for whole genomes [14]—, drastically decreasing the number of variants that have to run through any annotation pipeline. A data warehouse that incorporates sequencing results from thousands of individuals from various ethnic backgrounds and disease populations allows for fast cross-study analysis, such as differential mutation analyses, to discover novel genetic risk factors, gene–disease associations, potential disease mechanisms, and actionable variants [15–17]. The accumulated allele frequencies also help to gain an understanding of the distribution of disease-associated variants in reference populations.

Our second goal is to achieve a platform-independent solution, referring to data storage and computation infrastructure: relational databases, NoSQL, compute clusters, and Hadoop, each of which has its particular benefits for storage, indexing, querying, integration, or computation: some platforms are better suited to run secondary analysis pipelines and to call variants, some are better suited for computing allele frequencies across studies, some will be used to run graphical, interactive user interfaces, some to store and access summarized data, some to store per-individual data. We argue that such an endeavor requires a mechanism to compute a globally unique key for each normalized variant independently on each platform^1^. This will allow to easily map between every genetic variant resource employed across the entire infrastructure.

In summary, the functionality we present with the Reference Variant Store includes

- data from various large resequencing studies and annotation databases;
- extensive annotations including population frequencies, clinical significance, and predictions of functional impact;
- integrated analysis of disease versus healthy populations;
- a reversible variant key that uniquely identifies SNVs, MNVs, and indels, and that can be computed solely based on location and alleles;
- a RESTful web service to access bulk data programmatically; and
- per-sample information stored on Apache Hadoop allowing for fast computation of allele frequencies across populations, linkage disequilibrium, and population stratification.

We have so far populated RVS with variants from diverse resources shown in Table 1: RVS currently contains 473 million distinct variants at 389 million sites; 399 million of these variants have been observed in at least one of the studies we integrated; the remainder are largely hypothetical SNVs from dbNSFP^2^ [18].

**Table 1:** Number of variants imported from various external resources

Study	Variant sites	Variants	Unique to study	Variants passed	Samples
1000 Genomes [1]	81,195,126	81,693,252	57,400,612	all	2,504
ESP6500 [2]	1,982,177	1,998,204	184,225	all	6,503
UK10K [47] ALSPAC/TWINS	37,258,978	37,560,436	6,155,493	all	2,432
UK10K with disease ^c^	9,391,582	11,177,227	8,847,466	9,969,036	4,888
TCGA [4] germline ^c^	200,691,728	219,533,884	90,884,769	n/a	4,224
TCGA somatic	876,970	890,172	696,754	all	4,205
Scripps Wellderly [48]	76,144,271	91,947,469	63,331,143	53,303,437	534
ExAC ^b^ [3]	9,579,712	10,450,724	6,581,946	8,811,372	63,352
MSSM BioBank genotyping	849,806	849,806	0	all	11,210
In-house resequencing study	29,326,393	29,671,729	10,134,258	23,610,572	142
Total observed	358,152,122	399,404,510	244,216,666	>217,796,115	82,558 ^b^
Other resources:					
dbNSFP ^a^ [18]	30,523,109	89,617,785	73,561,239	—	—
ClinVar [12]	101,317	104,455	31,694	—	—
OMIM [49]	10,863	10,913	—	—	—
COSMIC [50]	1,483,983	1,525,243	—	—	—
PharmGKB ^c^ [51]	672	684	—	—	—
SwissVar ^d^	(77,047)	(84,649)	(34,198)	—	—
HGMD ^c^ [13]	125,744	133,464	32,178	—	—
Literature mining	—	890,665	—	—	—
Total observed + other	388,902,292	472,965,749	317,841,777	>217,796,115	82,558

The first block refers to sequencing/genotyping studies, the second to sample-independent annotation databases. “Unique to study” counts variants that were observed only in that particular study. “Variants passed” refers to variants that passed quality metrics as defined by the particular study, at least one sample has to pass; n/a: individual sample quality metrics not available. Totals exclude duplicates seen in different studies. Variants in annotation databases are included only if they can be mapped to precise coordinates and allele. Since a large proportion of the variants discovered by literature mining are given at the protein level only, they were not compared to other studies

^a^dbNSFP contains hypothetical variants, see text

^b^ExAC includes samples from 1000 Genomes, ESP6500, and TCGA

^c^Note that data from HGMD, PharmGKB, UK10K diseases and TCGA germline are not visible to external users on the RVS website

^d^Counts for SwissVar refer to distinct amino acid changes. Further details on individual resources are provided in Additional file 4: Table S3

Observed variants originate from 82,600 samples: 5,600 whole genomes, 66,000 whole exomes, and 11,000 genotyped samples. We also included variants that are annotated independent of samples, from resources such as ClinVar, OMIM, COSMIC, and the literature, adding to the observed and hypothetical variants.

The remainder of this paper is organized as follows. After presenting work closely related to ours, we shall provide details on the data sources and genetic variants imported into the Reference Variant Store so far, and show summary statistics as to variant types and impacts. We will then discuss applications and future directions for our work. We shall then explain the architecture and the workflows in RVS that support storage, annotation and loading of novel variants. We will lastly present details on the allele-specific variant key and literature mining.

### Related work

Several efforts share some of our goals in bringing together variants and annotations from large-scale sequencing studies. Chennagiri et al. [19] presented an idea to store genetic variants in a database for fast access, reduce redundancy, and Sanger benchmarking. They loaded more than 9000 samples from VCF files, including population frequencies from an early release of 1000 Genomes data clinical samples, and additional Sanger sequencing data. Annotations encountered in VCF files are stored as key–value pairs to support arbitrary tags. For RVS, we want to obtain (sub-) population frequencies, including disease population, from as many studies as possible. Clinical samples cover a variety of indications and originate from in-house and many external studies, genotyping and sequencing alike. We also enrich our annotations with by integrating renowned resources, such as ClinVar and OMIM.

CanvasDB^3^ is a local infrastructure supporting the analysis of resequencing projects, using MySQL for storage and providing an R interface for analysis [20]. As one major difference to RVS, CanvasDB stores the entirety of sample-specific genotypes, such as 1092 samples from the 1000 Genomes Project data. Users of CanvasDB can therefore perform SEQ-GWAS cohort analyses, defining cohorts on-the-fly and factoring in disease populations or family structures and the like. CanvasDB can be used as a fast and powerful filtering tool to analyze groups of samples. RVS aims at having data from several large cohort studies as well as various sources of annotation readily available for interpretation of observed variants.

GEMINI is a software package designed for exploring variation in personal genomes and family based genetic studies [21]. It utilizes resources such as KEGG and ENCODE for annotation of genes and ClinVar for variants. Once the local hosting solution is set up, users can import single samples or larger studies to store individual genotypes. Complex queries allow to find variants meeting different inheritance patterns, or run burden calculations. With RVS, in contrast, our focus is on providing detailed variant annotation on large numbers of preloaded variants and data from several large sequencing studies are readily available to the user; however, RVS currently does not store data by individual sample.

The Exome Aggregation Consortium recently presented their effort to make genetic variation data observed in 63,358 whole exomes publicly available [3]. ExAC brings together data from healthy and disease populations and can be searched by gene, variant, or dbSNP to show population frequencies and other annotations such as affected transcripts or disease association according to ClinVar. They also offer quality metrics to inform users about the reliability of calls, such as read depths histograms obtained from samples interrogated at each site. Contributing projects to date range from the 1000 Genomes and ESP to TCGA, Swedish Schizophrenia and Bipolar Studies, and several type 2 diabetes studies.

EVA, the European Variation Archive, collects highly detailed, granular, raw variant data from human (with other species to follow) [22, 23]. Types of genetic variation data include short as well as structural variations. EVA provides a web-based browser to query the entirety of variants for studies, genes, frequencies, and raw data, such as from VCFs. One of the benefits of EVA is that it allows users to submit variants obtained in their own studies by sample, supporting pedigree information as well. The focus of RVS in addition to the collection of variants lies on extensive annotation, in terms of population frequencies, clinical significance, predicted impacts, and so on.

The SG-ADVISER [24] is a standalone application that retrieves annotations for variants, including copy number, from a web-server on the fly. The back-end of SG-ADVISER utilizes a combination of precomputed data and high-performance computation on demand. Similar to RVS, the results include coding and protein impact, splicing impact, allele frequencies, and clinical annotations; in addition, data on regulatory variants, genomic regions, ontological information on processes, functions, and pathways are available.

## Construction and content

**Fig. 1:**
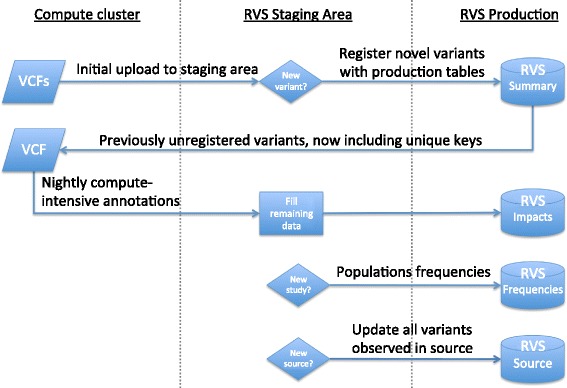
RVS architecture and workflow. All new variant data in VCF format gets populated into a staging area, where novel variants are registered with RVS. Novel variants are exported to the compute cluster for annotation with snpEff etc. Data are imported back into the production tables of RVS. Large studies will also trigger the upload of (sub)population frequencies. Variants in RVS are assigned to each new or updated source, allowing multiple sources per variant

The key components in the Reference Variant Store are *1)* a database infrastructure, *2)* pre-computed annotations for known genetic variants, *3)* insertion of novel variants from heterogeneous sources, and *4)* a unique ID to share data across platforms. Figure 1 shows an overview of the RVS architecture, depicting components for storage and computation, staging area, and import of new data. Table 2 and Additional file 1: Figure S1 show key tables in the production and staging areas. We will also describe our methodology to extract variants from the literature (PubMed abstracts and PubMedCentral full texts including supplementary files) in this section.

**Table 2:** Major tables in the Reference Variant Store that hold all imported variants and annotations

Table	Description
Summary	main table that stores each variant by chromosomal location, reference and alternate allele, dbSNP, and GRCh36/38 locations; most other tables are dependent tables
Impact	effect(s) on gene, transcript, intron/exon, missense/ non-sense, CDS and amino acid change, where applicable; by transcript
Frequencies	allele frequencies in large-scale sequencing studies (1000 Genomes, ESP6500, ExAC, Scripps Wellderly, etc.)
Predictions	computational predictions of functional impact, such as PolyPhen-2, MutationAssessor, SIFT, CADD, PROVEAN, GWAVA, and ensemble scores
Phenotypes	disease-associations from ClinVar, HGMD, OMIM, etc.
Regions	observed and predicted regions that contain the given variant: functional and regulatory elements (ENCODE), protein domains (InterPro), microRNA target sites (miRanda)
Source	maps each variant to the study/studies in which it was observed; also stores pass- or non-pass flags according to filtering criteria if provided by the study
Comments	*optional:* human expert comments on specific variants, pertaining to disease, impact, etc.
Staging_summary	registry that holds potentially new variants while they are not yet automatically annotated and copied to the production summary table
Staging_impact	holds results from computational models regarding effects of the mutation (protein level)

### Variant registry — summary, types, source

The main relational tables in the Reference Variant Store hold each observed variant using minimal information. The central summary table contains chromosome, start and end position, reference and alternate allele, variant type, dbSNP ID if available, size of the affected region, and the unique variant key. Coordinates currently default to GRCh37 and we hold the respective location on GRCh38 in addition. We also store a DB-internal, auto-increment variant ID and a numerical representation of the chromosome (X =23, Y =24, MT =25) for fast cross-referencing within the relational database only and for possible future partitioning of tables to increase performance. For each source of variants, we store the source study of each variant as a many–to–many lookup table.

### Variant annotation — impact, frequencies, predictions, phenotype, regions

We separate different basic types of annotations into multiple tables. *1)* Impacts are the immediate effects of a variant on gene, transcript, and protein sequences, such as amino acid change, frameshift, or promoter region, based on GRCh37 and ENSEMBL 78. Those can be computed using snpEff, ANNOVAR, and VEP [5–7]. We chose ENSEMBL and snpEff as our baseline and included additional annotations using snpEff on the RefSeq transcript models [25]. The infrastructure supports other annotation tools as well, see [26] for a detailed discussion. *2)* Predictions refer to the predicted functional impact of a variant, most often, on the protein level; computational tools include SIFT, PolyPhen-2, PROVEAN, MutationAssessor, CADD, GWAVA [8–10, 27–29], among others, and ensemble scores provided by dbNSFP [18]. *3)* We store allele frequencies pertaining to large sequencing efforts, such as 1000 Genomes, ESP6500, and Wellderly. *4)* If the variant is associated with a phenotype, such as a disease or risk factor for a disease, we provide this annotation together with its source, such as ClinVar and OMIM. *5)* Variants are annotated for occurrence in several kinds of DNA regions and protein domains: regulatory and functional regions from ENCODE [30]; predicted miRNA target sites [31]; and protein domains from InterPro [32]; *6)* In an optional table for comments, we hold annotations provided by in-house clinical experts on variants, particularly in the context of disease, to be displayed to users with appropriate privileges.

### Sample registry

RVS can optionally serve as a registry for studies and samples, without modifications to the variant registry and annotation tables. It is possible to also store genotype information per sample, including read depth, quality metrics, and so on; for our large-scale applications including hundreds of thousands of samples, however, individual information is stored in raw VCF files^4^ on Hadoop^5^. We update each imported VCF with the variant key, which can be computed independent of a central database lookup at any time (see later in this section for methods and limitations).

### Insertion of novel variants

The process of inserting variants into the Reference Variant Store is outlined in Fig. 1. We first load each variant into a staging area, from where we check if they already exist in RVS. If not, they will be registered in the production copy, to provide minimal information as obtained from the input (such as allele frequencies in the underlying study) or that are fast to compute (type of variant, effective size, variant key). Providers of new variants will therefore receive variant keys for their input in any case, for later reference. The variant will also be visible to queries against RVS right away, but devoid of detailed information as to transcripts, amino acid changes, etc. In nightly updates, which can also be triggered manually if required, we will then compute the left-normalized representation and provide missing annotations using snpEff.

### A globally unique, reversible identifier for small variants

Our Reference Variant Store is used not only to compute and related content inside a database instance, but to enable integration across compute and storage architectures. We therefore require a unique identifier, derived from the genomic location (genome build, chromosome, start, and end) and alternate allele. This *variant key* serves several purposes: *1)* for every new dataset, study or annotation, the key can be computed directly from the genomic location and alleles, thus there is no need for possibly expensive lookups in relational database tables or similar mechanisms; *2)* the key can be used completely independent from a central architecture to assign IDs to variants; *3)* when sharing results across platforms, databases tables, and so on, the key allows fast integration of data; *4)* the original genomic location and alleles can be computed from the variant key as a fallback and error recovery and to allow export; *5)* the key is a compressed version of the genomic coordinates and alleles; *6)* as an additional benefit, the variant key is valid across projects and alleviates data sharing on a larger scale.

The variant key we propose encodes the reference genome build (using GRCh version numbers), chromosome (1..22, X, Y, MT), start and end positions, and the alternate allele, in the following way: *1)* the first byte encodes the reference genome version, *2)* the second byte encodes the chromosome, *3)* bytes 3 to 7 store the start position, *4)* bytes 8 to 12 store the end position, *5)* bytes 13 and 14 store the length of the alternate allele (up to 4095), and *6)* all following bytes contain the compressed alternate allele. The encoding uses 64 characters in lexicographic order (digits, symbol ‘@’, upper case letters, symbol ‘_’, lower case letters) and therefore the variant key, when sorted, reflects the actual order of variants on the chromosome. The lexicographic order is useful for range queries as well, when using only the prefix of the variant key that denotes the assembly, chromosome, and start position.

The variant key is unique for SNVs, deletions, and for insertions/MNVs of up to 2958 inserted nucleotides. We decided on this restriction based on practical purposes, driven by data types and index key sizes in MySQL. We define small indels to be of effective size 1000 bp or less, referring to the absolute length difference of reference and alternate alleles. The latest release of 1000 Genomes Project data, for instance, contains 3283 indels of size larger than 1000. Those are included in RVS but have been assigned a potentially ambiguous variant key, and therefore should be accessed by chromosomal location and alt allele. We provide implementations in Python^6^, Java, JavaScript, Scala, and MySQL.

### Variants observed in the literature

We automatically extracted variants from the literature that are discussed with (mostly small-scale) sequencing efforts, genotyping, mutagenesis experiments, disease association, and other phenotypic impacts. The challenge in integrating variants from the literature with RVS is twofold: *1)* to detect genes and mutations with high precision (correct mutation, mapped to correct gene) and *2)* to convert each mutation into proper chromosomal location and alternate allele. If a variant is already contained in dbSNP, mapping it to a dbSNP entry helps solve *2)*. Otherwise, we need to deduce the chromosomal location from known coding sequences. In either case, we can end up with multiple transcripts, multiple locations, and multiple alternate alleles potentially underlying a reported amino acid change. To identify the correct reference genome build when chromosomal coordinates are given is also a challenge, since the build is not always explicitly mentioned in a publication.

We combined SETH ([33]; also see [34] for a comparison of recent tools) with GNAT [35] for the recognition of mutations and genes in text, respectively. SETH recognizes a variety of variants, such as single point mutations, indels, and structural variants, and attempts to map them to dbSNP and/or a protein sequence. It takes as input genes extracted and mapped to Entrez Gene by GNAT, as well as hand-curated data from NCBI’s gene2pubmed [36]. Jimeno Yepes et al. [34] compared the performance of several tools that recognize mutations in text and found that SETH outperformed several others on this task, with a precision of 89 at 68 % recall. SETH first converts every variant that it found into a canonical, or “grounded” form, similar to [37]. Grounded forms use the syntax suggested by the HGVS nomenclature [38], such as “c.396T >C” for a change in the coding sequence. SETH then compares each such variant to dbSNP, based on known CDS and protein changes and affected location and alleles, since dbSNP curates those annotations. For every variant that could not be mapped to dbSNP, SETH uses the protein sequence of genes found nearby to find the best match (if any). Since protein sequences may change over time but prior publications will seldom be corrected, we account for several causes underlying most discrepancies, such as omitted start codons and signal peptides, when matching an amino acid change onto a protein sequence by its position.

From 24 million MEDLINE^7^ citations and 3.1 million PubMed Central full texts^8^, we excavated more than 17 million occurrences of genetic variants (counting each individual occurrence in one single publication), see Table 3. We imported only those variants found in the literature that we were able to map to dbSNP or a gene/protein sequence, since only those would yield verifiable genomic coordinates and alleles. In PubMed, for instance, we found a total of 761,443 variants with evidence, 261,881 of which we successfully mapped to a dbSNP entry. Counting only unique alleles across three textual sources (Medline citation, PMC full text, supplementary files), RVS contains 890,665 alleles that we were able to map to one or more publications.

**Table 3:** Mutations extracted from PubMed/MEDLINE, PMC full texts, and PMC from PDFs including supplementary files such as Excel tables. Variants are grouped by variant type, counting each evidence for each variant resulting in the grand total. We also show the number of variants that we were able to map to a dbSNP ID, as well as the number of unique variants, disregarding occurrences across multiple publications

Type	PubMed	PMC	PDF and	Total
			Supplement	
Substitution	617,693	853,487	5,804,542	
dbSNP	102,040	222,310	4,433,018	
Insertion	3,072	2,252	17,640	
Duplication	875	1,263	5,522	
Repeat	42	76	339	
Deletion	19,987	27,192	69,326	
Insdel	202	290	2,061	
Frameshift	2,185	3,065	28,405	
Structural	15,347	6,143	5,642,341	
Total non-unique	761,449	1,116,093	15,802,854	
– with dbSNP ID	261,881	381,500	4,743,471	
Total unique	203,055	201,597	4,221,952	
Total unique mapped to allele ^a^	101,652	122,393	727,602	890,665

^a^In case amino acid changes were given in the literature, we counted only one allele that would lead to that change

Using a reverse-mapping of variants given as a HGVS CDS or protein change to possible transcripts, we compute the chromosomal location and alternate allele wherever possible, which is of most interest for variants without an apparent dbSNP entry. Using either the dbSNP ID or the chromosomal location, we can integrate variants identified by text-mining with the remainder of the Reference Variant Store and provide publications or text snippets as additional annotations. As mentioned in the Discussion, we are currently working on extracting disease–associations and other, lower level phenotypes such as changes in biochemical properties of proteins, from those textual evidences. We discuss our previous efforts to map variants to pharmacogenetic impacts in [39]. For now, RVS contains a simple mapping from a variant to the publications that it is described in, without further details on findings of the respective studies.

## Utility and discussion

RVS currently stores 399 million observed genetic variants at 358 million sites obtained from 82,600 samples, meaning each variant has been encountered in at least one sample. An additional 73 million hypothetical, single amino-acid-altering variants originating from dbNSFP enrich our existing annotations for potential future observations. Since we incorporated variants from clinical datasets such as ClinVar, COSMIC, and PharmGKB^9^, there are an additional approximately one million variants that have not been observed in one of the studies included in RVS thus far.

In total, RVS contains 473 million distinct genetic variants. Table 1 shows the current status of RVS in terms of the number of variants imported from external studies. In comparison, dbSNP build 141 contains 62.5 million RefSNP clusters for 261 million submissions. The latest release of 1000 Genomes Project data for Phase 3 v5 found 81.7 million variants at 81.2 million sites. As shown in Table 1, dbNSFP contains close to 90 million variants: namely, all hypothetical amino acid-changing, single-nucleotide variants. By combining currently ten cohorts, we find that 16 million of those variants (18 %) have actually been observed in at least one sample.

Using the GRCh37/ENSEMBL v78 transcript model [40], we mapped all 472 million variants to 1.955 billion impacts, where one variant typically maps to multiple transcripts (up to 5kb up- or downstream) and/or to an intergenic region. In addition, all variants were mapped to transcripts from RefSeq release 68 [25], smaller but more stable, resulting in 832 million impacts.

Among all variants observed in studies with ≥500 samples, 3.1 million variants hit a known InterPro protein domain. Additional file 2: Table S1 shows the number of variants per effect category on the transcript level, using one canonical transcript per variant and variants observed in a study with ≥500 samples. As canonical transcripts we define transcripts matching the canonical isoform provided by UniProt in protein sequence [41]. For each imported study we discard individual sample data and store only summarized information, such as allele and genotype frequencies. Additional file 3: Table S2 lists the amount of samples in RVS per technology, whole genome/exome sequencing or genotyping, and the typical number of base pairs covered in each.

It has to be noted that we import variants into RVS regardless of their validity as determined by the original study. Our main focus is on the annotation of each observed variant, whereas we decisively leave interpretation up to the user, as proper context is only known at the application level. Nevertheless, several of the studies we included in RVS provide quality metrics on a summarized or on an individual level, such as quality by read depth and average coverage across samples. When using the criteria for variants to pass defined by the respective study, 48.3 % of the variants reported by Scripps Wellderly, for example, have no individual sample that passed quality filters for this variant. In the UK10K disease cohorts we considered, the percentage of variants where at least one individual passed ranged from 44.5 to 78.9 %. Those quality metrics and cut-offs can differ widely. Quality metrics are not provided by each study, making it difficult to provide a final number of truly observed variants in RVS. Details on the number of variants before/after applying filters can be found in Table 1.

As one major driver behind integrating study data in RVS is to provide immediate access to precomputed annotations, we are also interested in how many new variants we can expect to find in a new study or individual sample. Table 1 shows the number of unique variants found in each original study, compared to all others in RVS, excluding hypothetical data in dbNSFP and annotations from ClinVar, HGMD, and literature mining. For whole genomes from 1000 Genomes project and TCGA germline, we observed that they add a comparable number of 22,900 and 21,500 unique variants on average per sample to RVS, respectively. The UK10K control data, despite similar sample size, seems to present with less unique variants in total and per sample—6 million in total, compared to 60 million in the 1000 Genomes, with about 2500 samples each.

### Distribution of clinically relevant variants

To get an idea about the distribution of clinically relevant variants in the population, we checked their respective allele frequency in RVS cohorts, emphasizing healthy cohorts. Table 4 shows the percentage of variants for each source and annotation that fall into a certain bin of allele frequencies. Allele frequencies were taken from 1000 Genomes Phase 3, ESP6500, Scripps Wellderly, UK10K ALSPAC/TWINS, and ExAC, for a total of 75,325 samples^10^. For each variant, we used the highest allele frequency found for any ethnicity (Additional file 4: Table S3 shows ethnicities for studies that had separate allele frequencies available). We excluded variants annotated with contradicting pathogenicities from different submitters to a source. For instance, some variants in ClinVar were annotated as both benign and pathogenic, with annotations originating from different publications. The total numbers of variants used to build Table 4 are 53,110 for ClinVar, 10,863 for OMIM, and 133,464 for HGMD.

**Table 4:** Variants in clinical annotation databases observed in healthy cohorts, binned by maximum ethnicity-specific allele frequency across cohorts. Bins are non-cumulative and intervals exclude the value of the upper boundary

Source	0	0–0.001	0.001–0.005	0.005–0.01	0.01–0.05	0.05–0.1	0.1–0.5	≥0.5	Total
ClinVar: pathogenic	30.09	2.59	0.86	0.20	0.26	0.05	0.14	0.02	34.21
ClinVar: likely pathogenic	3.26	0.29	0.08	0.01	0.02				3.66
ClinVar: risk factor	0.35	0.03	0.06	0.02	0.05	0.02	0.13	0.10	0.76
ClinVar: association	0.01	<0.01	<0.01	0.01	0.01	<0.01	0.05	0.02	0.10
ClinVar: likely benign	0.47	0.95	1.01	0.49	0.51	0.05	0.05	0.01	3.54
ClinVar: benign	0.49	0.26	0.47	0.62	2.33	1.10	2.61	1.70	9.58
ClinVar: protective	<0.01		<0.01			<0.01	0.02	0.01	0.03
ClinVar: drug response	<0.01				<0.01		0.01	0.01	0.02
ClinVar: uncertain significance	8.15	2.05	1.53	0.37	0.36	0.05	0.06	0.03	12.60
ClinVar: other	1.02	0.05	0.04	0.02	0.03	0.01	0.15	0.08	1.40
ClinVar: unknown	29.44	2.58	0.97	0.19	0.38	0.12	0.31	0.11	34.10
HGMD: DM	81.24	4.17	1.40	0.39	0.46	0.05	0.04	0.01	87.76
HGMD: DM?	4.80	0.61	0.50	0.17	0.35	0.12	0.17	0.02	6.74
HGMD: DFP	0.16	0.01	0.02	0.01	0.07	0.08	0.48	0.29	1.12
HGMD: DP	0.30	0.03	0.06	0.04	0.13	0.09	0.71	0.45	1.81
HGMD: FP	0.86	0.15	0.16	0.09	0.20	0.09	0.32	0.15	2.02
HGMD: FTV	0.32	0.06	0.03	0.01	0.03	0.01	0.04	0.03	0.53
OMIM: pathogenic	72.24	8.69	3.19	1.00	1.14	0.26	0.73	0.23	87.48
OMIM: probably pathogenic	0.02								0.02
OMIM: probably not pathogenic			0.01						0.01
OMIM: risk factor	1.33	0.23	0.28	0.06	0.29	0.13	0.59	0.57	3.48
OMIM: association	0.02	0.01		0.01		0.01	0.06	0.09	0.20
OMIM: no known pathogenicity	0.11	0.03	0.04	0.03	0.11	0.06	0.39	0.15	0.92
OMIM: confers sensitivity							0.01		0.01
OMIM: protective	0.01		0.01			0.03	0.06	0.05	0.16
OMIM: drug response		0.01			0.02		0.05	0.07	0.15
OMIM: other	6.46	0.32	0.15	0.06	0.03	0.03	0.04	0.01	7.10
OMIM: VUS	0.11	0.08	0.07	0.03	0.03	0.05	0.09	0.05	0.51

Values represent the percentage of variants from the respective resource that fall into each category and bin. DM, disease-causing mutation; DM?, likely DM; DP, disease-associated polymorphism; DFP, DP with additional functional evidence; FP, functional polymorphism; FTV, frameshift or truncating; VUS, variant of unknown significance

We found that 30 % of pathogenic variants in ClinVar did not occur in any of the RVS cohorts considered, as well as 72 % of OMIM variants and 81 % of HGMD disease causing mutations (DM). On the other hand, benign or protective variants tended to occur with higher frequencies. Note that those overall results were skewed to some extent by all-encompassing categories such as ‘unknown’ and ‘other’, as well as rare variants unlikely to be observed even among 75,325 individuals. In addition, the largest proportion of our data on those samples was produced using whole exome sequencing, omitting intronic and intergenic variants by design.

### Web query interface

**Fig. 2:**
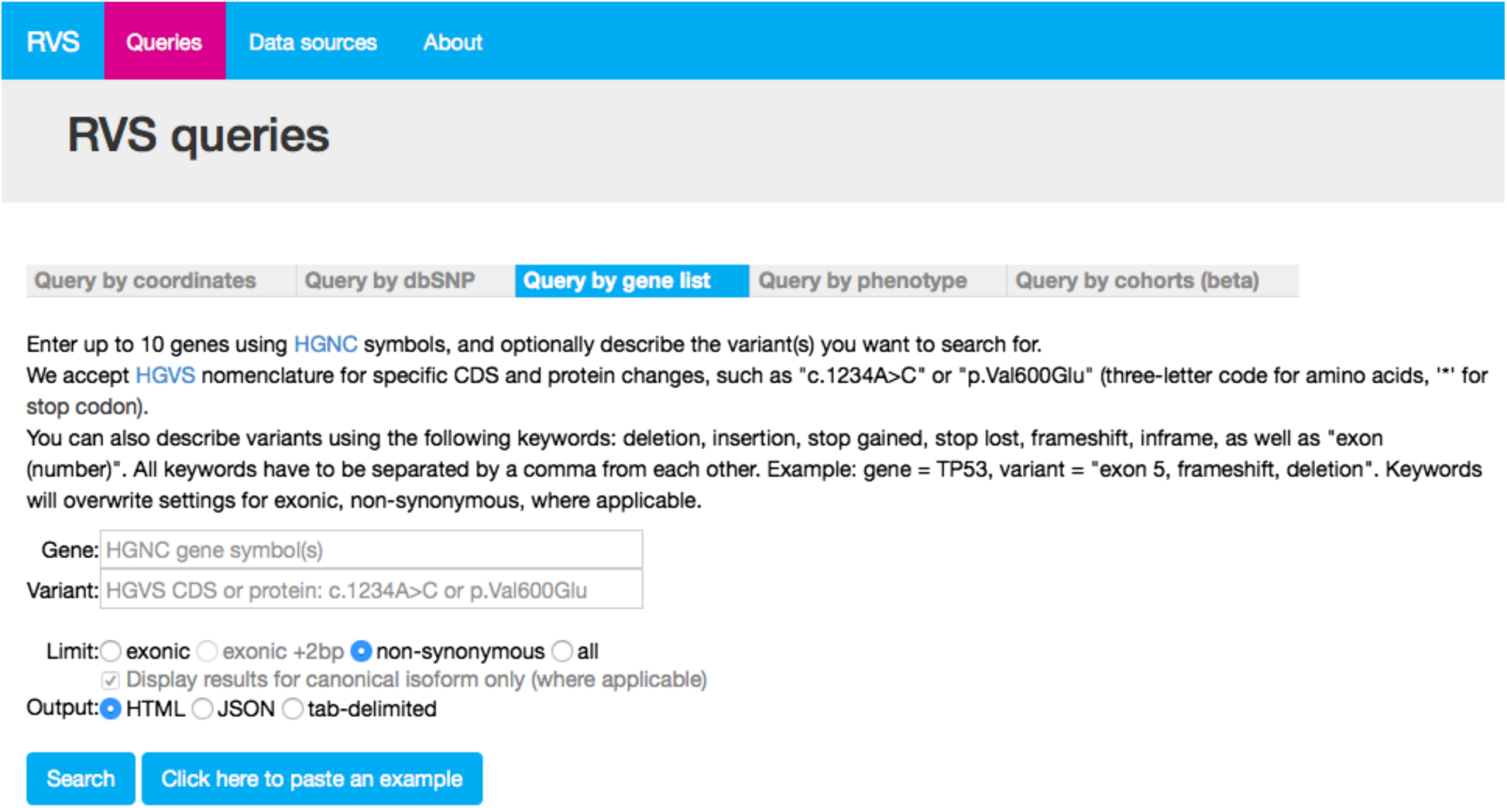
RVS web query interface: public datasets in RVS can be queried by coordinates (shown), dbSNP, genes, and by defining ‘cohorts’ using populations in RVS. RVS will return full annotations, frequencies, phenotypes, and literature references

We provide access to the public data sets using several search templates at https://rvs.u.hpc.mssm.edu/. Searches by gene, region, variant. or phenotype return all observed variants and respective allele frequencies. We also provide all annotations described in Methods and Table 2: transcripts, protein changes, predicted impacts from tools such as SIFT and MutationAssessor, associated phenotypes, and references to the literature. Another user query allows to compare different populations and return variants with significant differences in allele frequencies between the selected groups. Users can narrow variants down to those that hit exonic regions with or without splice sites, and to non-synonymous variants. Data can be exported in tab-delimited form and JSON. We show an example web query of RVS in Fig. 2. Results are organized by their source of information, such as basic information in the variant (location, allele, type, dbSNP membership); affected transcripts, effect, and resulting amino acid changes; population frequencies; and references to databases and literature.

### RESTful web service

To accommodate batch queries, RVS accepts REpresentational State Transfer (REST) requests to obtain data for different resource types, namely population frequencies, impacts such as protein changes, computational predictions, and associated phenotypes. Supported arguments are gene, chromosomal location, dbSNP ID, phenotype, and variant key. This allows users to fetch all population frequencies (a resource) for a given dbSNP ID (an argument), for instance. We limited requests to one resource and one type of argument per call. To implement batch queries, users can send individual requests for each variant, with up to ten chromosomal locations and/or regions at a time, and obtain annotations. Users can do the same for up to ten dbSNP IDs or ten genes as well. Results are returned in JavaScript Object Notation (JSON) to support nested data, such as predicted impact scores applied to different amino acid positions, which depend on the transcripts that overlap a requested chromosomal location. Optional arguments allow to specify filters on the results, such as returning information only on the canonical transcript (if any), variants that results in an amino acid change, or variants that have been observed in a sequencing study; as opposed to hypothetical variants from dbNSFP, or variants annotated in ClinVar but not seen in any of our reference populations.

### Population frequencies of variants in ClinVar

As a possible application of RVS, we are working on validation of suspected disease variants in various annotation databases, including ClinVar, HGMD, and GAD. Guidelines have been brought forward as to assess causality of variants in human disease [42]. As one first step, researchers should meticulously check available data for subpopulation-specific allele and carrier frequencies, which RVS provides. ClinVar, for example, holds genetic variants related to human health and annotates them as pathogenic, likely pathogenic, risk allele, or benign (among others). Assuming that no pathogenic variant should occur with considerable frequency in any healthy population, we can use the information accumulated in RVS to cross–check allele frequency against tens of thousands of samples with known ethnicity. We set a threshold of 1 % allele frequency in any population, although 0.1 % would be an acceptable stricter alternative for autosomal dominant disorders. 349 variants in ClinVar have an allele frequency of ≥ 1 *%* in either 1000 Genomes or ESP6500 (total or by super-population). Out of those, 195 variants are annotated as “pathogenic” in ClinVar, with an additional eight “likely pathogenic” variants. 80 out of 349 are already annotated as “benign” or “likely benign”, with the remainder being of uncertain significance, protective, or having a mixture of annotations.

### Identifying disease causing variants

Another application of RVS is the identification of potential disease causing variants. Those can be variants that are observed exclusively in disease populations (heterozygous, unaffected carriers may exist) and that have a likely functional impact. RVS is particularly well suited to compare genotype frequencies across any number of healthy and disease populations. The annotations that we load for each variant provide information as to its impact on the protein level: variant affecting splice sites, producing early termination codons, and so on. Loss-of-function variants are of particular interest to the research community in analyzing causality in disease [42].

We already imported several disease cohorts into RVS, many with appropriate controls provided within the same study. Since allele frequencies are preloaded for each of the larger studies (hundreds of samples), we can quickly discover variants possibly implicated in some rare diseases, for example from UK10K samples, by comparing allele frequencies. To assess sensitivity and specificity of this methodology, we can compare such results with data already published on the respective original study, for example, [43, 44]. Excluding, for example, variants that have also been observed in the 1000 Genomes Project, ESP6500, Scripps Wellderly, UK10K ALSPAC/TWINS, and variants observed in other rare diseases from UK10K, we can re-discover variants such as MAB21L2 c.152G >A, which were unknown prior to the UK10K data release and their initial publications. For the Coloboma eye disease data, we found a total of 88 variants that follow those criteria (no carriers in healthy or other disease populations).

Since RVS aims at incorporating as many observed variants as possible for computing their impact, it also includes low quality variants. We store information regarding the quality metrics as supplied by each study; at the lowest common level, these would flag whether or not a variant passed the filtering criteria suggested by the respective study^11^. Such information should be considered when trying to identify or validate disease causing variants.

To achieve results of higher quality, aforementioned analyses also need to incorporate ancestry information on each individual. Where not available, we are experimenting with inferring the ethnicity of an individual using principal component analysis (PCA) and ancestry informative markers (AIMS).

### Future directions

Future directions we are pursuing are the integration of disease populations, inclusion of structural variants, text mining for functional consequences, cloud storage for public access, and transition to GRCh38, among others.

A full transition of RVS to GRCh38, while keeping GRCh36 and GRCh37 locations for fast integration with legacy data, is our immediate next step. Since GRCh38 incorporates numerous haplotypes (alternate loci, currently in 178 regions), a focus will be on the design of a unique identifier for build 38 that can capture this variation.

As a related issue, the current design handles only short insertions and multi-nucleotide variants, limited to about 3000 bp, while deletions are unlimited in size. This is due to a technical limitation of the underlying database and its maximum index size. RVS does not at this point store copy number variation, gene fusion events, or other larger-scale structural rearrangements, as well as loss of heterozygosity, all of which we aim to include in future releases. Those data will particularly boost applications of RVS in oncology research, where a large number of such events have been observed in past and ongoing sequencing projects. One current drawback of such data is that precise coordinates are often not known, so we would require a mechanism to match imprecise regions with each other and specific short variants.

We are also contemplating to store the data, once preprocessed, on cloud services such as Amazon. It will then be available to the research community running Hadoop/EMR applications that build on top of the information we provide, without requiring the invocation of web services, and without the need for each individual group to replicate and maintain such data. One issue in this regard are the different access models (data usage agreements, licenses), which need to be considered. At the current stage, we are not providing certain data from PharmGKB and HGMD, as well as TCGA germline and UK10K disease cohorts publicly on the RVS website.

In many cases, the functional consequences of a specific variant are not known. We are currently mapping more than 4.5 million variants that we detected in the literature to experimental findings, such as a change in protein function, gene expression, or drug resistance, to provide those annotations with RVS. This effort is a continuation of our prior work on detection of genetic variants in text and their association with diseases and drug response phenotypes [33, 39]. We are now focusing on changes in biochemical properties of the DNA, mRNA, and protein, for which experiments such as mutagenesis, have been carried out and results reported in the literature.

In addition to functional scoring methods for coding changes, we will also incorporate predictions on aberrant splicing, such as the destruction of known sites favoring cryptic splice sites, or mutations occurring within exonic and intronic splice site enhancing and silencing regions.

As a final building block in RVS, we are currently incorporating public and non-public data from disease cohorts, such as GERA [45], ADNI [46], WGS500^12^, and the dbGaP Compilation of Individual-Level Genomic Data for General Research Use (GRU)^13^ into a joined Disease Variant Store (DIVAS). This will add another level of annotation to variants, for phenotypes and disease population frequencies, and allow for more powerful analyses across studies that are currently hindered by small sample sizes and limited genetic background. An integration of data collected on similar phenotypes in distinct studies, for instance, while at the same time providing data on healthy individuals, can help glimpse into (rare) disease-causing variants and their mechanisms.

## Conclusions

We presented here our implementation of a reference variant store (RVS). RVS hosts germline, somatic, and hypothetical genetic variants from large sequencing and genotyping studies, including the 1000 Genomes Project, ESP6500, UK10K, Scripps Wellderly, and TCGA. We store the precomputed effect (affected gene, transcript, protein), impact (functional predictions), population frequencies (healthy and disease), and disease association with experimental evidence (such as ClinVar and literature mining) as annotations for each variant. In total, RVS consist of over 470 million genetic variants thus far, representing 78,500 samples. Overall, we found 244 million variants that were unique to a single study, out of 400 million observations made in one or more studies; these numbers exclude hypothetical data from dbNSFP and sample-independent clinical databases such as ClinVar. Our two main goals are first to provide quick turn-around times for the full annotation of individually sequenced genomes^14^; and second to support exploratory analyses across all studies. As such, RVS facilitates cross-study analysis to discover novel genetic risk factors, gene–disease associations, potential disease mechanisms, and actionable variants. Due to its large reference populations, RVS can also be employed for variant filtration and gene prioritization, providing allele frequencies in healthy populations, integrated with protein-level annotations and known disease-associations.

Detected variants are submitted to RVS, which returns unique variant keys that can also be computed independent of centralized lookup tables, on any computational platform. Truly novel variants will be annotated on-the-fly or during nightly builds, whereas annotations for previously encountered variants are available immediately. We envision that with each newly added dataset and individual genome, the burden of computing effect and impact of new variants will become less until having to add only a minimal amount of variants for each newly sequenced individual. For example, it has been shown that the number of novel SNVs per genome rapidly drops from an initial 3,500,000 variants in the first whole genome to less than 150,000 new variants after assessing the twentieth genome [14]. We found that after having stored about 5,000 whole genomes, each new genome on average adds just over 3,000 new variants.

Every new annotation dataset that we load into RVS, and that contains annotations per variant or per gene, for disease associations, functional impact, pharmacogenetics, etc., can be easily extended by adding the unique variant key, allowing for immediate integration with existing variant calls and propagation of the data to our tools and search interfaces. This setup also allows for frequent updates of the underlying disease-association databases without having to re-annotate VCF files and/or relational tables holding per-sample data.

## Availability

A web interface to public datasets and annotations in RVS is available at https://rvs.u.hpc.mssm.edu/.

## Endnotes

^1^ Referring to left-aligned variants, in gene regions described using HGVS nomenclature; see https://github.com/counsyl/hgvs and [37, 38].

^2^ dbNSFP consists of all hypothetical single nucleotide variants that would result in an amino acid change, including stop lost and gained, and holds predicted impact scores.

^3^ CANdidate Variant Analysis System and Data Base: https://github.com/UppsalaGenomeCenter/CanvasDB

^4^ VCF format: http://www.1000genomes.org/wiki/Analysis/Variant%20Call%20Format/vcf-variant-call-format-version-41

^5^ Apache Hadoop: http://hadoop.apache.org/

^6^ A Python package for normalizing variants and generating variant keys is available at https://github.com/weiyi-bitw/varnorm

^7^ Medline: http://www.ncbi.nlm.nih.gov/pubmed/

^8^ PMC: http://www.ncbi.nlm.nih.gov/pmc/

^9^ Note that we included variants from clinical datasets only if precise coordinates and alleles were available, therefore not storing variants referred to as “del 5kb” and similar occurrences.

^10^ We decided not to use TCGA germline allele frequencies, due to uncertain genotypes that are devoid of homozygous alternate calls in all but breast cancer and some kidney chromophobe data.

^11^ Note that filtering criteria may vary widely between studies. Not all information necessary to apply our own metrics are consistently available to us.

^12^ WGS500: http://www.well.ox.ac.uk/wgs500

^13^ GRU: http://www.ncbi.nlm.nih.gov/projects/gap/cgi-bin/collection.cgi?study_id=phs000688

^14^ By extension, whole exome sequencing, other targeted sequencing, genotyping arrays.

## Additional files

Additional file 1
**Database schema of RVS.** Supplementary figure 1 summarizes the database schema of RVS production tables; some columns were omitted, such as detailed prediction scores and columns for sub-population frequencies. For detailed explanations of tables, refer to Table 2. (PDF 164 kb)

Additional file 2
**Variants in RVS by type (silent, frameshift, etc.).** Supplementary table 1 shows the effects of observed variants. Shown are the numbers of variants that fall into a specific category of the SequenceOntology (http://www.sequenceontology.org/), as determined by snpEff [5]. Counts are based on observations in studies with ≥500 samples, unfiltered, and take into account one canonical transcript per variant. Regions up/downstream of a gene are limited to 5000 bp. Effects with less than 10 matching variants are omitted. (PDF 18.8 kb)

Additional file 3
**Sequencing cohorts in RVS.** Supplementary table 2 summarizes the sample cohorts in RVS by sequencing/genotyping technology: Approximate number of base pairs covered; targeted regions in whole exome sequencing depend largely on the capturing kit, see [52] for an overview. (PDF 15.1 kb)

Additional file 4
**Data sources integrated in RVS.** Supplementary table 3 provides details on major external resources integrated into RVS. (PDF 16.3 kb)

## References

[1] Abecasis GR, Altshuler D, Auton A, Brooks LD, Durbin RM, Clark AG (2010). A map of human genome variation from population-scale sequencing. Nature.

[2] Tennessen JA, Bigham AW, O’Connor TD, Fu W, Kenny EE, Gravel S (2012). Evolution and functional impact of rare coding variation from deep sequencing of human exomes. Science.

[3] Exome Aggregation Consortium (2014). Exome Aggregation Consortium (ExAC).

[4] TCGA Research Network. The Cancer Genome Atlas. 2014. http://cancergenome.nih.gov/, last access on Dec 1, 2015.

[5] Cingolani P, Platts A, Wang leL, Coon M, Nguyen T, Wang L (2012). A program for annotating and predicting the effects of single nucleotide polymorphisms, SnpEff: SNPs in the genome of Drosophila melanogaster strain w1118; iso-2; iso-3. Fly (Austin).

[6] Wang K, Li M, Hakonarson H (2010). ANNOVAR: functional annotation of genetic variants from high-throughput sequencing data. Nucl Acids Res.

[7] McLaren W, Pritchard B, Rios D, Chen Y, Flicek P, Cunningham F (2010). Deriving the consequences of genomic variants with the Ensembl API and SNP Effect Predictor. Bioinformatics.

[8] Kumar P, Henikoff S, Ng PC (2009). Predicting the effects of coding non-synonymous variants on protein function using the SIFT algorithm. Nat Protoc.

[9] Adzhubei I, Jordan DM, Sunyaev SR (2013). Predicting functional effect of human missense mutations using PolyPhen-2. Curr Protoc Hum Genet.

[10] Choi Y, Chan AP. PROVEAN web server: a tool to predict the functional effect of amino acid substitutions and indels. Bioinformatics. 2015. 10.1093/bioinformatics/btv195 PMC4528627 25851949

[11] Sherry ST, Ward MH, Kholodov M, Baker J, Phan L, Smigielski EM (2001). dbSNP: the NCBI database of genetic variation. Nucl Acids Res.

[12] Landrum MJ, Lee JM, Riley GR, Jang W, Rubinstein WS, Church DM (2014). ClinVar: public archive of relationships among sequence variation and human phenotype. Nucl Acids Res.

[13] Stenson PD, Ball EV, Mort M, Phillips AD, Shaw K, Cooper DN (2012). The Human Gene Mutation Database (HGMD) and its exploitation in the fields of personalized genomics and molecular evolution. Curr Protoc Bioinforma.

[14] Pelak K, Shianna KV, Ge D, Maia JM, Zhu M, Smith JP (2010). The characterization of twenty sequenced human genomes. PLoS Genet.

[15] Mooney SD, Krishnan VG, Evani US (2010). Bioinformatic tools for identifying disease gene and SNP candidates. Methods Mol Biol.

[16] Sarkar IN, Butte AJ, Lussier YA, Tarczy-Hornoch P, Ohno-Machado L (2011). Translational bioinformatics: linking knowledge across biological and clinical realms. J Am Med Inform Assoc.

[17] Dewey FE, Grove ME, Pan C, Goldstein BA, Bernstein JA, Chaib H (2014). Clinical interpretation and implications of whole-genome sequencing. JAMA.

[18] Liu X, Jian X, Boerwinkle E (2013). dbNSFP v2.0: a database of human non-synonymous SNVs and their functional predictions and annotations. Hum Mutat.

[19] Chennagiri N, Breton B, Umbarger M, Saunders P, Porreca G, Kennedy C (2013). A generalized scalable database model for storing and exploring genetic variations detected using sequencing data. ASHG Annual Meeting.

[20] Ameur A, Bunikis I, Enroth S, Gyllensten U (2014). CanvasDB: a local database infrastructure for analysis of targeted- and whole genome re-sequencing projects. Database (Oxford).

[21] Paila U, Chapman BA, Kirchner R, Quinlan AR (2013). GEMINI: integrative exploration of genetic variation and genome annotations. PLoS Comput Biol.

[22] Lappalainen I, Spalding D, Saha S, Skipper L, Ameida-King J, Kumanduri V, et al. European Variation Archive. 2014. http://www.ebi.ac.uk/eva. last accessed 06/2015.

[23] Lappalainen I, Lopez J, Skipper L, Hefferon T, Spalding JD (2013). DbVar and DGVa: public archives for genomic structural variation. Nucl Acids Res.

[24] Erikson GA, Deshpande N, Kesavan BG, Torkamani A (2015). SG-ADVISER CNV: copy-number variant annotation and interpretation. Genet Med..

[25] Pruitt KD, Brown GR, Hiatt SM, Thibaud-Nissen F, Astashyn A, Ermolaeva O (2014). RefSeq: an update on mammalian reference sequences. Nucl Acids Res.

[26] McCarthy DJ, Humburg P, Kanapin A, Rivas MA, Gaulton K, Cazier JB (2014). Choice of transcripts and software has a large effect on variant annotation. Genome Med.

[27] Reva B, Antipin Y, Sander C (2011). Predicting the functional impact of protein mutations: application to cancer genomics. Nucl Acids Res.

[28] Kircher M, Witten DM, Jain P, O’Roak BJ, Cooper GM, Shendure J (2014). A general framework for estimating the relative pathogenicity of human genetic variants. Nat Genet.

[29] Ritchie GR, Dunham I, Zeggini E, Flicek P (2014). Functional annotation of noncoding sequence variants. Nat Methods.

[30] Dunham I, Kundaje A, Aldred SF, Collins PJ, Davis CA, Doyle F (2012). An integrated encyclopedia of DNA elements in the human genome. Nature.

[31] Betel D, Wilson M, Gabow A, Marks DS, Sander C (2008). The microRNA,org resource: targets and expression. Nucleic Acids Res.

[32] Mitchell A, Chang HY, Daugherty L, Fraser M, Hunter S, Lopez R (2015). The InterPro protein families database: the classification resource after 15 years. Nucleic Acids Res.

[33] Thomas P, Rocktäschel T, Mayer Y, Leser U. SETH: SNP Extraction Tool for Human Variations. 2014. http://rockt.github.io/SETH/, last access on Dec 1, 2015.

[34] Jimeno Yepes A, Verspoor K (2014). Mutation extraction tools can be combined for robust recognition of genetic variants in the literature. F1000Res.

[35] Hakenberg J, Gerner M, Haeussler M, Solt I, Plake C, Schroeder M (2011). The GNAT library for local and remote gene mention normalization. Bioinformatics.

[36] Brown GR, Hem V, Katz KS, Ovetsky M, Wallin C, Ermolaeva O (2015). Gene: a gene-centered information resource at NCBI. Nucl Acids Res.

[37] Hart RK, Rico R, Hare E, Garcia J, Westbrook J, Fusaro VA (2015). A Python package for parsing, validating, mapping and formatting sequence variants using HGVS nomenclature. Bioinformatics.

[38] den Dunnen JT, Antonarakis SE (2000). Mutation nomenclature extensions and suggestions to describe complex mutations: a discussion. Hum Mutat.

[39] Hakenberg J, Voronov D, Nguyen VH, Liang S, Anwar S, Lumpkin B (2012). A SNPshot of PubMed to associate genetic variants with drugs, diseases, and adverse reactions. J Biomed Inform.

[40] Flicek P, Amode MR, Barrell D, Beal K, Billis K, Brent S (2014). Ensembl 2014. Nucl Acids Res.

[41] Bateman A, Martin MJ, O’Donovan C, Magrane M, Apweiler R, Alpi E (2015). UniProt: a hub for protein information. Nucl Acids Res.

[42] MacArthur DG, Manolio TA, Dimmock DP, Rehm HL, Shendure J, Abecasis GR (2014). Guidelines for investigating causality of sequence variants in human disease. Nature.

[43] Chandra A, Arno G, Williamson K, Sergouniotis PI, Preising MN, Charteris DG (2014). Expansion of ocular phenotypic features associated with mutations in ADAMTS18. JAMA Ophthalmol.

[44] Rainger J, Pehlivan D, Johansson S, Bengani H, Sanchez-Pulido L, Williamson KA (2014). Monoallelic and biallelic mutations in MAB21L2 cause a spectrum of major eye malformations. Am J Hum Genet.

[45] Hoffmann TJ, Kvale MN, Hesselson SE, Zhan Y, Aquino C, Cao Y (2011). Next generation genome-wide association tool: design and coverage of a high-throughput European-optimized SNP array. Genomics.

[46] Mueller SG, Weiner MW, Thal LJ, Petersen RC, Jack CR, Jagust W (2005). Ways toward an early diagnosis in Alzheimer’s disease: the Alzheimer’s Disease Neuroimaging Initiative (ADNI). Alzheimers Dement.

[47] Kaye J, Hurles M, Griffin H, Grewal J, Bobrow M, Timpson N (2014). Managing clinically significant findings in research: the UK10K example. Eur J Hum Genet.

[48] Scripps Wellderly Genome Resource. The Scripps Wellderly Study. 2014. ftp://stsi-ftp.sdsc.edu/pub/wellderly/. last accessed 03/2015.

[49] Amberger JS, Bocchini CA, Schiettecatte F, Scott AF, Hamosh A (2015). OMIM,org: Online Mendelian Inheritance in Man (OMIM *®*;), an online catalog of human genes and genetic disorders. Nucl Acids Res.

[50] Forbes SA, Beare D, Gunasekaran P, Leung K, Bindal N, Boutselakis H (2015). COSMIC: exploring the world’s knowledge of somatic mutations in human cancer. Nucl Acids Res.

[51] Thorn CF, Klein TE, Altman RB (2013). PharmGKB: the Pharmacogenomics Knowledge Base. Methods Mol Biol.

[52] Chilamakuri CS, Lorenz S, Madoui MA, Vodak D, Sun J, Hovig E (2014). Performance comparison of four exome capture systems for deep sequencing. BMC Genomics.

