# Supplementary figures and images for: Integrating 400 million variants from 80,000 human samples with extensive annotations: towards a knowledge base to analyze disease cohorts
Source: BMC Bioinformatics. 2016 Jan 8;17:24. doi: 10.1186/s12859-015-0865-9 (PMC4706706; doi:10.1186/s12859-015-0865-9)

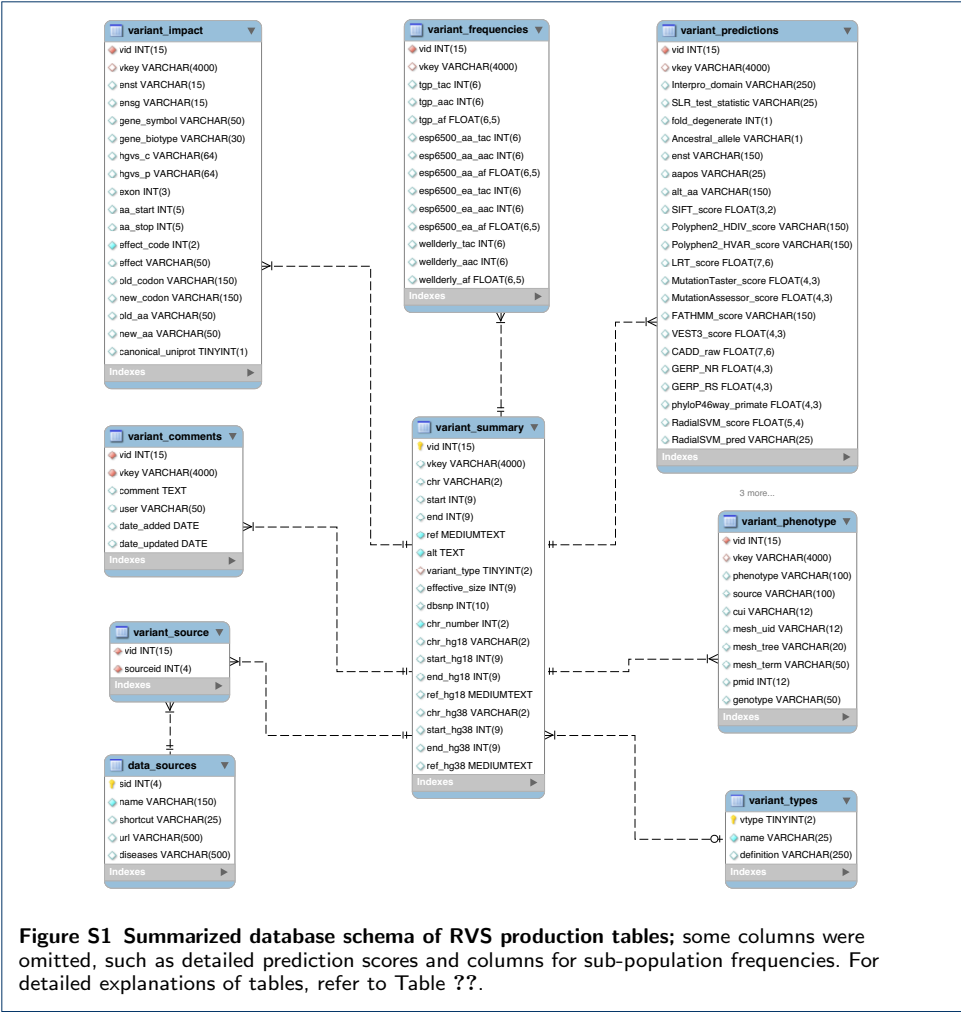

Supplement: Additional file 1 — Database schema of RVS. Supplementary figure 1 summarizes the database schema of RVS production tables; some columns were omitted, such as detailed prediction scores and columns for sub-population frequencies. For detailed explanations of tables, refer to Table 2. (PDF 164 kb) [file 12859_2015_865_MOESM1_ESM.pdf]
