# Supplementary material for: Integrating 400 million variants from 80,000 human samples with extensive annotations: towards a knowledge base to analyze disease cohorts
Source: BMC Bioinformatics. 2016 Jan 8;17:24. doi: 10.1186/s12859-015-0865-9 (PMC4706706; doi:10.1186/s12859-015-0865-9)
Supplement: Additional file 2 — Variants in RVS by type (silent, frameshift, etc.). Supplementary table 1 shows the effects of observed variants. Shown are the numbers of variants that fall into a specific category of the SequenceOntology (http://www.sequenceontology.org/), as determined by snpEff [5]. Counts are based on observations in studies with ≥500 samples, unfiltered, and take into account one canonical transcript per variant. Regions up/downstream of a gene are limited to 5000 bp. Effects with less than 10 matching variants are omitted. (PDF 18.8 kb) [file 12859_2015_865_MOESM2_ESM.pdf]

Table S1 Effects of observed variants. Shown are the numbers of variants that fall into a specific category of the SequenceOntology (http://www.sequenceontology.org/), as determined by snpEff [?]. Counts are based on observations in studies with $\geq$500 samples, unfiltered, and take into account one canonical transcript per variant. Regions up/downstream of a gene are limited to 5000bp. Counts for intergenic regions are not shown in this table. Effects with less than 10 matching variants are omitted. '+' indicates that a variant falls into multiple categories, such as an inframe deletion that leads to the loss of a stop codon.

Effect	Count	Effect	Count
intron_variant	103,440,930	frameshift_variant+missense	999
downstream_gene_variant	10,171,427	stop_lost	740
upstream_gene_variant	9,346,371	exon_loss_variant	695
3'UTR_variant	3,368,125	stop_gained+inframe_insertion	474
synonymous	2,292,056	stop_gained+disruptive_inframe_deletion	332
frameshift	1,385,311	stop_gained+disruptive_inframe_insertion	309
5'UTR_variant	1,056,181	frameshift_variant+synonymous	219
missense_variant	802,216	initiator_codon_variant	176
splice_region_variant	518,745	missense_variant+inframe_deletion	149
5'UTR_premature_start_codon_gain	180,894	stop_lost+inframe_deletion	134
non_coding_exon_variant	163,722	missense_variant+disruptive_inframe_deletion	104
inframe_deletion	23,257	start_lost+inframe_deletion	69
splice_acceptor_variant	22,903	exon_loss_variant+synonymous_variant	44
splice_donor_variant	21,675	frameshift_variant+stop_gained+missense	42
stop_gained	20,495	exon_loss_variant+start_lost+synonymous	41
disruptive_inframe_deletion	16,857	stop_lost+disruptive_inframe_deletion	24
inframe_insertion	13,387	missense_variant+inframe_insertion	23
frameshift_variant+stop_gained	9,007	missense_variant+disruptive_inframe_insertion	19
disruptive_inframe_insertion	6,060	exon_loss_variant+stop_lost+synonymous	18
stop_retained_variant	2,068	start_lost+disruptive_inframe_insertion	13
frameshift_variant+stop_lost	1,820	start_lost+inframe_insertion	12
start_lost	1,446	start_lost+disruptive_inframe_deletion	11
frameshift_variant+start_lost	1,230	stop_gained+inframe_deletion	10
