# Supplementary material for: Integrating 400 million variants from 80,000 human samples with extensive annotations: towards a knowledge base to analyze disease cohorts
Source: BMC Bioinformatics. 2016 Jan 8;17:24. doi: 10.1186/s12859-015-0865-9 (PMC4706706; doi:10.1186/s12859-015-0865-9)
Supplement: Additional file 3 — Sequencing cohorts in RVS. Supplementary table 2 summarizes the sample cohorts in RVS by sequencing/genotyping technology: Approximate number of base pairs covered; targeted regions in whole exome sequencing depend largely on the capturing kit, see [52] for an overview. (PDF 15.1 kb) [file 12859_2015_865_MOESM3_ESM.pdf]

Table S2 Samples in RVS by sequencing/genotyping technology. Approximate number of base pairs covered. *Targeted regions in whole exome sequencing depend largely on the capturing kit, see [?] for an overview.

Technology	Samples in RVS	Base pairs
Whole genome sequencing	5,612	>3 billion
Whole exome sequencing*	65,736	51-64 million
Genotyping	11,210	<1 million
