# Supplementary material for: Integrating 400 million variants from 80,000 human samples with extensive annotations: towards a knowledge base to analyze disease cohorts
Source: BMC Bioinformatics. 2016 Jan 8;17:24. doi: 10.1186/s12859-015-0865-9 (PMC4706706; doi:10.1186/s12859-015-0865-9)
Supplement: Additional file 4 — Data sources integrated in RVS. Supplementary table 3 provides details on major external resources integrated into RVS. (PDF 16.3 kb) [file 12859_2015_865_MOESM4_ESM.pdf]

**Table S3 Details on major external resources integrated into RVS. *TCGA uses self-reported ethnicities.**

| Study | Version | Samples | Ethnicities | Reference |
|---|---|---|---|---|
| 1000 Genomes | Phase 3, v5.20130502 | 2,504 | Five major groups: African, Ad Mixed American, East Asian, South Asian, European; 26 subpopulations | [?] |
| ESP6500 | V2 SSA137 (dbSNP 138) | 6,503 | African American, European American | [?] |
| ExAC | Release 0.3 | 63,352 | African/African American, American, East Asian, Finnish, Non-Finnish Europeans, South Asian | [?] |
| TCGA* | | 4,224 | African, American Indian, Asian, Hispanic/Latino, Native Hawaiian, White | [?] |
| dbNSFP | v2.5 | — | — | [?] |
| ClinVar | Release 2015-02-03 | — | — | [?] |
| COSMIC | v71 10-21-14 | — | — | [?] |
| SwissVar | Downloaded: 2015/03 | — | — | [?] |
| HGMD | 2015.1 | — | — | [?] |
| ENSEMBL | v78 | — | — | [?] |
| RefSeq | Release 68 | — | — | [?] |
